# Differences in the palliative care phase between patients with nonmalignant pulmonary disease and lung cancer: a retrospective study

**DOI:** 10.1186/s12904-024-01618-w

**Published:** 2024-12-26

**Authors:** Hanna Pihlaja, Heidi A. Rantala, Silja Soikkeli, Milja Arminen, Sonja Aho, Sirpa Leivo-Korpela, Juho T. Lehto, Reetta P. Piili

**Affiliations:** 1 Faculty of Medicine and Health Technology, Tampere University, Arvo Ylpön katu 34, Tampere, 33520 Finland; 2 Palliative Care Centre, Tampere University Hospital, Tampere, Finland; 3 Department of Respiratory Medicine, Tampere University Hospital, Tampere, Finland; 4 Cancer Centre, Department of Oncology, Tampere University Hospital, Tampere, Finland

**Keywords:** Palliative care, Specialist palliative care, Nonmalignant pulmonary disease, Lung cancer, Survival, Care arrangements, Emergency room, Hospitalization

## Abstract

**Background:**

Patients with chronic nonmalignant pulmonary disease and lung cancer both need palliative care, but palliative care services may be better adjusted to serve cancer patients. We compared the timing and clinical practice of palliative care and acute hospital usage during the last year of life in patients with nonmalignant pulmonary disease or lung cancer.

**Methods:**

This was a retrospective study of all patients in a palliative care phase (palliative goal of care) with nonmalignant pulmonary disease or lung cancer who were treated at Tampere University Hospital, Finland, during the years 2018–2020. The data were collected from the hospital’s medical records. Comparisons between the groups were performed by using the Pearson chi-square test, Fisher’s exact test, or Mann‒Whitney U test when appropriate. Survival was estimated by using the Kaplan‒Meier method.

**Results:**

The study population consisted of 107 patients with nonmalignant pulmonary disease and 429 patients with lung cancer. Patients with nonmalignant pulmonary disease survived longer in the palliative care phase than patients with lung cancer (115 vs. 59 days, *p* < 0.001). Compared to lung cancer patients, patients with nonmalignant disease received a palliative care specialist consultation more often during hospitalization (66% vs. 45%, *p* < 0.001) than during a preplanned outpatient visit (6% vs. 52%, *p* < 0.001), were less likely to be referred to palliative care pathway (79% vs. 87%, *p* = 0.033), and spent more days in an acute care hospital during the last year of life (median of 10 vs. 6 days, *p* = 0.023). Contrary to lung cancer patients, referral to the palliative care pathway was not significantly associated with decreased acute hospital resource usage during the last month of life among patients with nonmalignant pulmonary disease.

**Conclusions:**

Compared to lung cancer patients, patients with nonmalignant pulmonary disease had longer palliative care phases but fewer visits to the palliative care outpatient clinic and fewer referrals to the palliative care pathways. Palliative care arrangements seemed to have more influence on the end-of-life care of lung cancer patients. There is a need for long-term palliative care services with better abilities to meet the special needs of patients with nonmalignant pulmonary disease.

## Background

Patients with nonmalignant pulmonary disease and lung cancer experience similar symptom burdens at the end of life (EOL), including breathlessness, pain, anxiety, depression, and fatigue [1–4]. Thus, high-quality palliative care services should be offered for both patient groups [5].

Palliative care for advanced respiratory disease can be accomplished at many levels of proficiency depending on the needs of the patient, and early integration of palliative care for the treatment of patients is recommended [5, 6]. Palliative care may last for months- or even a few years before the final EOL care is needed during the last weeks or days of life [7]. However, recognizing the need for the palliative goal of care and referral to palliative care services may be challenging, especially in patients with nonmalignant pulmonary disease with a long and variable disease trajectory [8, 9].

In Finland, physicians are recommended to identify patients with very advanced disease and a need to shift the goal of care to a palliative intent by making a so-called palliative care decision together with the patient and his/her closest ones [10]. This is documented with the International Classification of Diseases (ICD-10) code Z51.5 Palliative Care in the patient records. According to national guidelines, all Finnish physicians are recommended to make these decisions as a part of advance care planning when appropriate and to consult or refer their patients to specialist palliative care services when needed [7, 10].

Palliative care arrangements have been traditionally developed to serve cancer patients, and the benefits of timely palliative care decisions have been shown in patients with cancer [11, 12]. Although physicians may recognize the deterioration and palliative care needs of patients suffering from nonmalignant pulmonary disease, it remains unclear whether palliative care decisions have a similar timing and impact on EOL care in patients with nonmalignant pulmonary disease as they do in patients with cancer. Sorting out potential differences between these patient groups on the practices of decision-making, and palliative care delivered to patients after changing the goal of care might provide valuable information for the development of palliative care services.

The aims of this study were to compare the timing and clinical practice of palliative care and the use of hospital resources during the last year of life in patients with nonmalignant pulmonary disease or lung cancer.

## Methods

### Design

We performed a retrospective study of all patients with a nonmalignant pulmonary disease or lung cancer and a documented palliative care decision (ICD-10 code Z51.5) treated at Tampere University Hospital between January 1, 2018, and December 31, 2020. Patients were followed up until death or December 31, 2021.

Approval for this study and permission to access patient records were obtained from the Pirkanmaa Hospital District, Tampere, Finland (R20592). According to Finnish law and research regulations, no ethics committee approval was needed for this retrospective register-based study. This study was conducted according to national laws, regulations, and the Declaration of Helsinki.

### Setting

Tampere University Hospital provides tertiary-level healthcare services for a population of 900,000 inhabitants and secondary-level services for 530,000 inhabitants in the Pirkanmaa region. The hospital’s palliative care unit provides specialist palliative care through an outpatient clinic and a palliative care consultation team. The collaboration between hospice, different hospital wards, outpatient clinics, and primary palliative care is fluent. The palliative care unit provides approximately 1,000 inpatient consultations and 1,200 outpatient visits a year. The multidisciplinary team of the palliative care unit includes physicians with special competency in palliative medicine, nurses with special training in palliative care, and other professionals when needed.

Palliative care decision (ICD-10 code Z51.5) is defined as setting the goal of care to symptom-centered palliative care among patients with very advanced diseases, whose survival cannot be markedly prolonged with disease-centered therapies, or the patient does not prefer them [7]. The decision is made in agreement with the physician, patient, and his/her closest ones. In this article, the palliative care phase refers to the time between the palliative care decision and death. Aggressive life-prolonging therapies, such as chemotherapy in cancer patients, are considered futile and discontinued or ruled out after the palliative care decision is made [11]. Simultaneously, in patients with nonmalignant pulmonary disease, aggressive therapies, such as lung transplant or invasive ventilation, are no longer considered an option when making a palliative care decision. In addition, at the time of this decision, the disease typically causes suffering and a need for more intensive support for patients or their caregivers. The physician responsible for the decision suggests that the disease trajectory is worsening and arrangements for the EOL are needed closely in the future. Palliative care decisions (ICD-10 code Z51.5) are recorded annually for more than 1500 patients at the Tampere University Hospital.

The regional palliative care pathway consists of regional home care teams, community hospitals, and one hospice (the Pirkanmaa hospice). Patients included in the pathway should have a palliative care decision and thereafter they may be admitted to a community hospital ward or hospice directly without needing a visit to the emergency room (ER). In addition, EOL care at home until death is arranged when feasible.

### Participants

A total of 1347 patients with a diagnosis of nonmalignant pulmonary disease or lung cancer and a documented palliative care decision (ICD-10 code Z51.5) were identified from the Tampere University Hospital patient records. After excluding patients who had another advanced disease as the primary reason for the palliative care decision, 536 patients with chronic nonmalignant pulmonary disease or lung cancer were included in this study.

### Data collection

All the hospital’s electronic medical records were reviewed. The collected data included age, sex, main diagnosis indicating the palliative care decision, living conditions, smoking status, pack-years, comorbidities, and the date of death. Patients were considered to need help in activities of daily living (ADL) if they were supported by a caregiver at home (a family member or home care services) or were permanently accommodated in a nursing home or community hospital. The Charlson comorbidity index (CCI) was calculated for each patient [13, 14].

We recorded specialist palliative care outpatient visits and consultations provided by the hospital’s palliative consultation team and evaluated the details of the palliative care decision by collecting the date and place of the decision. Do not attempt to resuscitate (DNAR) orders and decisions to withhold intensive care (e.g. admission to intensive care unit (ICU) and invasive mechanical ventilation) were also recorded.

Referral to the palliative care pathway was reported if the patient was directed to the local physicians and nurses in the communities and preplanned EOL care by the home care team, primary care ward, or a hospice was organized for the patient.

For the last year of life, we recorded all ER visits and acute hospitalization days (i.e., preplanned follow-up visits were excluded) at the Tampere University Hospital.

### Data analysis

Given that most of the distributions were nonnormal, the nonparametric Mann‒Whitney U test was used for continuous variables. The Pearson chi-square test or Fisher’s exact test was used for categorical variables when appropriate. Survival estimations were made by the Kaplan‒Meier method with the log-rank test.

Statistical significance was set as a p-value less than 0.05. Analyses were performed using IBM SPSS Statistics version 27.0 (IBM Corp. Armonk, NY, 2020).

## Results

Patient characteristics are presented in Table 1. Of the study population, 107 patients had nonmalignant pulmonary disease (58% chronic obstructive pulmonary disease (COPD), 40% interstitial lung disease), and 429 patients had lung cancer. Of the patients with lung cancer, 144 (34%) also had a diagnosis of COPD. A greater proportion of patients with nonmalignant pulmonary disease needed help with ADLs, even though the CCI was higher among patients with lung cancer.

**Table 1:** Patient characteristics

	Total		Nonmalignant pulmonary disease		Lung cancer		*p*-value
Total number, n (%)	536	(100)	107	(20)	429	(80)	
Male, n (%)	335	(63)	57	(53)	278	(65)	0.028
Age, median years (range)	74	(36–98)	77	(60–92)	74	(36–98)	< 0.001
Needing help with ADL, n (%)^a^	390	(73)	90	(84)	300	(70)	0.001
Smoking status, n (%)							0.063
Never-smoker	85	(15.9)	25	(23.4)	60	(14.0)	
Ex-smoker	322	(60.1)	62	(57.9)	260	(60.6)	
Current smoker	124	(23.1)	19	(17.8)	105	(24.5)	
Unknown	5	(0.9)	1	(0.9)	4	(0.9)	
Pack-years, median (range)^b^	40	(1-100)	40	(2–80)	40	(1-100)	0.342
Charlson comorbidity index, median (range)	9	(2–16)	6	(2–12)	10	(4–16)	< 0.001
Dead by the end of follow-up	510	(95)	93	(87)	417	(97)	< 0.001

ADL, activities of daily living

^a^ Missing value in 2 patients due to loss of data in patient records

^b^ Missing value in 27 patients due to loss of data in patient records. Never-smokers excluded

**Fig. 1:**
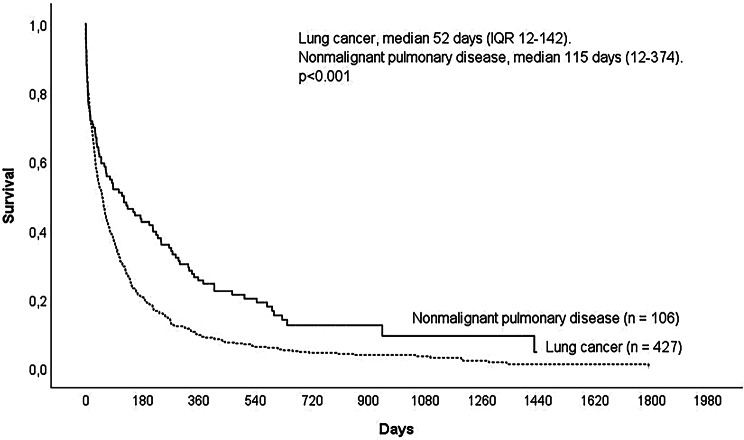
Survival after the palliative care decision in patients with lung cancer or nonmalignant pulmonary diseases. For 3 patients, data were not available

By the end of follow-up, 93 (87%) of the patients with nonmalignant pulmonary disease and 417 (97%) of the patients with lung cancer had died. A greater proportion of patients with nonmalignant pulmonary disease survived longer than one year compared to patients with lung cancer (25% vs. 10%, *p* < 0.001). The median survival times from the palliative care decision were 115 days (IQR 12–374) and 52 days (IQR 12–142) in patients with nonmalignant pulmonary disease and lung cancer, respectively (*p* < 0.001) (Fig. 1).

The characteristics of the palliative care services and treatment decisions of the patients are presented in Table 2. Patients with lung cancer visited the palliative care outpatient clinic more often than patients with nonmalignant pulmonary disease did (52% vs. 6%, *p* < 0.001). For patients with nonmalignant pulmonary disease, contact with specialist palliative care services occurred mainly through consultation during hospitalization. Palliative care decisions were made most often in an outpatient setting for patients with lung cancer, whereas decisions were made in the hospital ward for more than half of the patients with nonmalignant pulmonary disease. Specialists from the palliative care unit participated in the discussion and decision-making process leading to palliative care decision more often with patients with nonmalignant pulmonary disease than patients with lung cancer (59% vs. 34%, *p* < 0.001, respectively). Patients with nonmalignant pulmonary disease were less likely referred to the palliative care pathway than patients with lung cancer (79% vs. 87%, *p* = 0.033). In both patient groups, the palliative care unit’s participation increased the number of patients taken into the palliative care pathway (67% vs. 90%, *p* < 0.001). Patients with nonmalignant pulmonary disease more often had decisions to withhold intensive care.

**Table 2:** Characteristics of the palliative care services and treatment decisions in patients with nonmalignant pulmonary disease and lung cancer

	Total		Nonmalignant pulmonary disease		Lung cancer		*p*-value
All patients, n (%)	536	(100)	107	(20)	429	(80)	
Patients receiving specialist palliative care, n (%)	391	(73)	74	(69)	317	(74)	*0.324*
Patients visiting palliative care outpatient clinic, n (%)	230	(43)	6	(6)	224	(52)	*< 0.001*
Patients receiving palliative care team consultation, n (%)	265	(49)	71	(66)	194	(45)	*< 0.001*
Patients referred to palliative care pathway, n (%)	456	(85)	84	(79)	372	(87)	*0.033*
Place of palliative care decision, n (%)							*0.006*
Ward	249	(46)	64	(60)	185	(43)	
Outpatient clinic	235	(44)	30	(28)	205	(48)	
Intensive care unit	5	(1)	2	(2)	3	(1)	
Other^a^	47	(9)	11	(10)	36	(8)	
Patients having DNAR order, n (%)	525	(98)	107	(100)	418	(97)	*0.132*
Patients having a decision to withhold intensive care, n (%)	352	(66)	85	(79)	267	(62)	*< 0.001*

^a^ Decisions made at palliative home care, primary care, or emergency room

DNAR, do not attempt resuscitation

Table 3 presents the association of palliative care pathway referrals on the utilization of healthcare resources during the last month of life. Referral to the palliative care pathway was significantly associated with a decreased number of ER visits and acute hospitalization days during the last month of life in patients with lung cancer but not in patients with nonmalignant pulmonary disease.

No significant difference was found in the number of visits to the ER during the last month or year of life between the patients with lung cancer and those with nonmalignant pulmonary disease overall, but patients with nonmalignant disease had more acute hospitalization days during the last year of life (median of 6 vs. 10 days, *p* = 0.023). Fifty-seven (14%) patients with lung cancer and 17 (18%) with nonmalignant pulmonary disease died at Tampere University Hospital (*p* = 0.254).

**Table 3:** The association of palliative care pathway referrals on the utilization of healthcare resources during the last month of life in patients with nonmalignant pulmonary disease or lung cancer

	Total		Referred to the pathway		Not referred to the pathway		*p*-value
Number of patients, n (%)^a^							
Lung cancer	417		361	(87)	56	(13)	
Nonmalignant disease	93		73	(78)	20	(22)	
Patients visiting ER < 1 month before death, n (%)							
Lung cancer	142	(34)	112	(31)	30	(54)	< 0.001
Nonmalignant disease	37	(40)	28	(38)	9	(45)	0.591
Acute inpatient days < 1 month before death, median (range)							
Lung cancer	0	(0–31)	0	(0–31)	2	(0–26)	*0.002*
Nonmalignant disease	0	(0–31)	0	(0–28)	0	(0–31)	*0.753*

ER, emergency room

^a^ death by the end of follow-up

## Discussion

We showed that patients with nonmalignant pulmonary disease had longer survival in the palliative care phase than patients with lung cancer, and their specialist palliative care consultations occurred mostly during acute hospitalization. Patients with lung cancer visited the palliative care outpatient clinic more often, and the palliative care pathway had more effect on the use of acute hospital resources compared to patients with nonmalignant pulmonary disease.

The longer survival in the palliative care phase in patients with nonmalignant pulmonary disease compared to those with cancer challenges the planning for EOL care. In patients with nonmalignant pulmonary disease, the disease trajectories vary and are difficult to predict, and advanced disease is usually characterized by several acute exacerbations before final EOL care is needed [8, 15]. This may be a barrier for both patients and physicians in engaging in advance care planning and palliative care [16–20]. Furthermore, there is a risk that patients repeatedly contact acute care services instead of palliative care providers during the long palliative care phase if palliative care follow-up visits are not organized regularly.

In this study, contact with specialist palliative care in patients with nonmalignant pulmonary disease occurred mostly through consultations during acute exacerbation when it can be challenging to assess the patient’s prognosis and future palliative care needs. Wysham et al. also reported that patients with nonmalignant disease were more likely than cancer patients to have their initial palliative consultation at the intensive care unit and less likely as an outpatient [3]. Specialist consultation during an exacerbation may concentrate mostly on symptom relief, and after surviving an exacerbation, the actual referral to palliative care services may be missed. Previous studies suggest that the reasons for the lack of referral to palliative care services include uncertainty about the prognosis, fear of taking away the patient´s hope, lack of knowledge about palliative care, and that palliative care is falsely understood as a synonym for EOL care [16–18, 21–23]. Thus, more referrals and routine follow-up visits, phone calls, or video visits to palliative care units should be promoted for patients with nonmalignant pulmonary disease in the future to ensure the continuation of palliative care also after surviving an exacerbation. This continuation of care would also ensure that approaching EOL is recognized, and patients can be referred to EOL services well in advance.

The prognosis of patients with advanced lung cancer has changed due to the new landscape of cancer therapeutics, and cancer patients are now coping with greater uncertainty regarding their disease trajectory [6]. However, patients with advanced disease still seem to have poor survival if the disease leads to hospitalization shortly after diagnosis or if the disease progresses despite oncologic therapies leading to the palliative care phase [24]. In this study, the median survival of the lung cancer patients after the palliative care decision was only 52 days. Our results highlight the need for rapid access to palliative care services, including EOL care arrangements, for patients with lung cancer after changing the goal of care into palliative intent. In addition, patients with lung cancer probably benefit from early integration of palliative care and advance care planning visits to palliative outpatient clinics, where the focus can be addressed to symptom control and psychological support early enough [24–26].

Intake in a palliative care pathway was associated with a significant reduction in the number of ER visits and hospitalization days during the last month of life among patients with lung cancer, but this was not found among patients with nonmalignant pulmonary disease. Our results are in line with the recent study of McIagan et al., where palliative care did not reduce the use of hospital resources among COPD patients [27]. In our population of patients living in rural areas, palliative care pathways often rely on EOL care organized in primary palliative care, such as community hospital wards. Healthcare professionals working in primary palliative care may be more familiar with the symptoms and disease trajectory of cancer patients than patients with nonmalignant pulmonary disease. We hypothesize that the competency of primary palliative care may be insufficient for some patients with advanced nonmalignant pulmonary disease to take care of difficult symptoms, such as dyspnea. Dyspnea is known to be a common trigger for ER visits and hospitalization among palliative patients with nonmalignant pulmonary disease [28, 29]. Therefore, in the future, we should be more prepared to increase the intensity of palliative care for patients with nonmalignant pulmonary disease if necessary. This could include e.g. the use of high-flow nasal therapy or noninvasive mechanical ventilation (in order to relieve symptoms), which are not usually available at primary care hospitals or home care [30, 31]. Also, palliative sedation may be needed in some patients with nonmalignant pulmonary disease at EOL especially due to refractory dyspnea [32]. The ability for this around the clock may be only accomplished at specialist palliative care. In conclusion, in addition to primary palliative care, we need more specialist palliative care wards and home care teams to take care of the most challenging situations to avoid burdensome ER visits and admissions to acute hospitals for patients with nonmalignant pulmonary diseases.

Interestingly, patients with nonmalignant disease did not die in a secondary hospital despite the use of acute hospital services during the last month of life. Apparently, patients were taken care of at primary palliative care after ER visits or acute hospitalization, but as discussed above, avoiding this use of burdensome acute care in the first place should be prevented more effectively in the future.

Most of the patients with nonmalignant pulmonary disease needed help with ADLs, and they spent more time in the hospital during the last year of life compared to cancer patients. Considering the long palliative care phase of these patients, the need for help can last for several years. Supporting patients’ formal and informal caregivers and offering skillful home care as a part of palliative care is of utmost importance and has also recently recommended [5, 17, 33]. The fragility of patients with nonmalignant pulmonary disease may also be one of the reasons behind the low number of outpatient visits since they might be too burdensome in the palliative phase of the illness. This highlights the importance of the availability of home-delivered palliative care. The higher CCI score of the lung cancer patients than of the patients with nonmalignant pulmonary disease despite their lower need for ADLs is explained by the high points given by metastatic tumors in the CCI.

In this study, all patients with nonmalignant pulmonary disease had a DNAR order recorded, and most of them had a decision to withhold intensive care. These rates were higher than in patients with lung cancer and higher than in a study conducted by Raskin et al., in which 61% of patients with COPD had a DNAR order recorded before death [34]. In Finland, discussions about limits of care are seen as important, and it is known that in Northern Europe, DNAR and do-not-intubate orders are more frequently recorded during hospitalization than in Southern Europe [35].

Additionally, in patients with nonmalignant pulmonary disease, discussions about withholding resuscitation and intensive care might be seen as more important since the possibility of intubation and invasive mechanical ventilation in acute exacerbations are also present in the early palliative care phase, and the poor prognosis of these intensive treatments in patients with advanced disease are seen beforehand [36]. In contrast, in patients with lung cancer, respiratory insufficiency usually develops just before death during the EOL, when life-sustaining treatments are no longer an option. According to previous studies, the likelihood of invasive or noninvasive ventilation and cardiopulmonary resuscitation is greater in patients with nonmalignant pulmonary disease than in patients with lung cancer [15, 37]. Furthermore, the older age of patients with nonmalignant pulmonary disease likely increases the number of decisions to limit care [34]. It is also known that palliative care contact increases the number of these decisions [38]. Beyond DNAR and a decision to withhold admission to intensive care units, there are other limitations to care, such as avoiding hospitalizations or antibiotics, that can be addressed in the patient records. However, in this data, the numbers of these markings were so few and rather unclearly expressed that we did not include them in the analyses.

### Limitations

These were real-life data, and information relevant to this study was possible to retrieve retrospectively. A small sample size limits the statistical power, which may not have been sufficient to detect weaker associations in statistical analyses. This must be taken into account especially when interpreting our results on the relatively small number of patients with nonmalignant pulmonary disease. Since this was a single-center study from Finland, the generalizability of the results to other nations must be done with caution.

We collected data only from the patient records of Tampere University Hospital; thus, we lacked information on the use of other healthcare resources and places of death. On the other hand, we aimed to examine the role of specialist palliative care, and the information necessary for our results could be gathered at the university hospital level.

Although national recommendations instruct physicians to recognize patients in a palliative care phase by recording diagnosis code Z51.5 in patient records, we assume that particularly patients with nonmalignant pulmonary disease remained underrepresented in this study because of the lack of this diagnosis code. Thus, we are not able to describe the care of the patients with possible unrecognized need for palliative care during the last year of life in our hospital. Nevertheless, the use of this diagnosis code as a search criterion gives us a unique opportunity to specifically examine patients with advanced disease and a recognized palliative phase of the illness, where suitable palliative care arrangements should be equally available for all patients.

## Conclusions

Palliative care services seemed to be more adjusted to serve cancer patients than patients with nonmalignant pulmonary disease in our population. Patients with nonmalignant pulmonary disease had longer palliative care phases but fewer visits to the palliative outpatient clinic and referrals to the palliative care pathway. Despite palliative care arrangements, they frequently visited the ER and were hospitalized. The special needs in symptom control and complex disease trajectory of patients with nonmalignant pulmonary disease should be noted since the practices of care may fall beyond what we have learned when taking care of cancer patients. Future studies should examine the most appropriate palliative care delivery models for patients with nonmalignant pulmonary disease.

## Data Availability

The data can be requested from the corresponding author.

## Abbreviations

EOL: End of life
ADL: Activities of daily living
CCI: Charlson comorbidity index
COPD: Chronic obstructive pulmonary disease
ER: Emergency room
DNAR: Do not attempt to resuscitate
